# A novel *lamin A/C* gene mutation causing spinal muscular atrophy phenotype with cardiac involvement: report of one case

**DOI:** 10.1186/s12883-015-0269-5

**Published:** 2015-02-20

**Authors:** Naotoshi Iwahara, Shin Hisahara, Takashi Hayashi, Jun Kawamata, Shun Shimohama

**Affiliations:** Department of Neurology, School of Medicine, Sapporo Medical University, South 1 West 16, Chuo-ku Sapporo, 060-8543 Japan; Department of Pharmacology, School of Medicine, Sapporo Medical University, South 1 West 16, Chuo-ku Sapporo, 060-8543 Japan

**Keywords:** Spinal muscular atrophy (SMA), *lamin A/C* (*LMNA*), Atrioventricular block, Laminopathy, Cardiomyopathy

## Abstract

**Background:**

Mutations of the *lamin A/C* gene have been associated with several diseases such as Emery-Dreifuss muscular dystrophy, dilated cardiomyopathy and Charcot-Marie-Tooth disease, referred to as laminopathies. Only one report of spinal muscular atrophy and cardiomyopathy phenotype with *lamin A/C* gene mutations has been published. The concept that *lamin A/C* gene mutations cause spinal muscular atrophy has not been established.

**Case presentation:**

We report a man aged 65 years who presented with amyotrophy of lower limbs, arrhythmia and cardiac hypofunction. He showed gait disturbance since childhood, and his family showed similar symptoms. Neurological and electrophysiological findings suggested spinal muscular atrophy type 3. Gene analysis of *lamin A/C* gene showed a novel nonsense mutation p.Q353X (c.1057C > T). Further investigations revealed that he and his family members had cardiac diseases including atrioventricular block.

**Conclusions:**

We report the first Japanese case of spinal muscular atrophy phenotype associated with *lamin A/C* mutation. When a patient presents a spinal muscular atrophy phenotype and unexplained cardiac disease, especially when the family history is positive, gene analysis of *lamin A/C* gene should be considered.

## Background

Spinal muscular atrophy (SMA) is classified as a lower motor neuron disease, and is caused by degeneration of neurons in the anterior horn of the spinal cord. SMA is clinically classified into five subtypes based on onset age and progression [1,2]. In Japan, most of the patients with SMA types 1 and 2 have deletions or mutations of the *survival motor neuron* gene (*SMN1*), while only one-half of the patients with SMA type 3 have *SMN1* deletions or mutations [3], and other responsible genes are rarely identified.

Since Sterz et al. [4] reported SMA type 3 with disturbed cardiac rhythm in two brothers, only a few reports described cardiac involvement in SMA. Most of the reported cases demonstrated atrial arrhythmia and/or a variable degree of atrioventricular block. Additionally, some cases had cardiac dysfunction with ventricular and atrial dilatations commonly seen in dilated cardiomyopathy (DCM) [5]. The genetic factor underlying this combined phenotype had remained unknown until Rudnik-Schöneborn et al. [6] reported *lamin A/C* gene (*LMNA)* mutations in families with adult-onset SMA and cardiac disease.

We report the first Japanese case of SMA with cardiac diseases associated with *LMNA,* and a novel nonsense mutation p.Q353X (c.1057C > T).

## Case presentation

A 65 year-old Japanese man attended for the first time to Sapporo Medical University Hospital, and was admitted because of gait disturbance and amyotrophy of lower limbs. No perinatal abnormalities were observed, except that he had been enable to walk until three years old. He was clumsy in running since childhood and walking became gradually more difficult with age. He had attended to other neurological hospital in his 40s having given no reasonable diagnosis, stopped seeing doctors. He began to use a walker two years before admission. He had a history of atrial fibrillation at 43 years of age and complete atrioventricular block at 45 years, after which a pacemaker was implanted at other hospital. He had hypertension since the age of 57, and had two attacks of cerebral embolism in the last decade.

**Figure 1:**
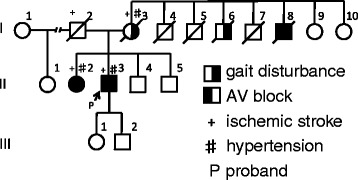
**Pedigree of the patient’s family.** Arrow indicates the patient as II-3. The genetic analysis was limited to the proband, as we could not get a permission from other family members.

The pedigree of his family is shown in Figure 1. His mother (I-3), two uncles (I-6, 8) and sister (II-2) had gait disturbance, and his sister (II-2) also had a pacemaker implanted due to atrioventricular block. However, no detailed information on disease onset and progression was available in their medical records. His sister also had gait disturbance since childhood and atrioventricular block was detected in early adulthood. She had more severe muscle weakness than the patient, and was unable to get up by herself. She could not come to our hospital because of her physical condition, and rejected to get a genetic analysis. His mother and sister had hypertension and ischemic stroke. His children were in their 30’s and remained healthy with no gait disturbance and cardiac disease. Our investigation found no consanguineous marriage.

On admission, his heart rate was 60 rpm (pacemaker rhythm), and blood pressure was 119/75 mmHg while on antihypertensive drugs. Whereas transthoracic echocardiography showed diffuse cardiac hypofunction with ejection fraction 42%, no ventricular and atrial dilatations were observed. According to his medical record, his ejection fraction was 62% in his 50’s and had deteriorated gradually.

A neurological examination revealed significant muscle weakness in lower limbs with *pes cavus.* The extent of muscle weakness was the same in the proximal and distal parts of lower limbs (Table 1). Muscle weakness in upper limbs and swallowing impairment were mild (Table 1). Mild joint contracture was observed in his right knee joint. Muscle weakness was more prominent on the left side because of ischemic stroke (Table 1). Fasciculation was observed in both legs. His tendon reflexes were absent. There were no abnormalities in sensory and autonomic systems. Evaluation of cognitive function showed mild cognitive impairment, with Mini-Mental State Examination score of 23/30 and Frontal Assessment Battery score of 12/18.

**Table 1:** **Medical Research Council Scale and needle electromyograms of the patient**

	**MRC**		**nEMG**
	**Lt**	**Rt**	**Lt**
Neck extension	5		NE
Neck flexion	4		NE
Dorsal flexion	4		
(Th10 paraspinal muscle)			Chronic neurogenic change
Deltoid	5	5	NE
Biceps	4	4	Chronic neurogenic change
Triceps	5	5	NE
Wrist extension	3	5	NE
Wrist flexion	5	5	NE
Iliopsoas	4	4	NE
Gluteus medius	3	3	NE
Quadriceps	3	3	Chronic neurogenic change
Hamstrings	3	3	NE
Tibialis anterior	3	4	Chronic neurogenic change
Gastrocnemius	2	3	Chronic neurogenic change

MRC: Medical Research Council Scale, nEMG: needle electromyograms, NE: not examined.

Examinations of blood and cerebrospinal fluid showed no remarkable abnormalities, and creatine kinase was slightly high (179 IU/L: normal value 45–160 IU/L). The patient refused a muscle biopsy. A brain computed tomography scan revealed multiple old cerebral infractions, but hippocampal atrophy was not indicated.

**Figure 2:**
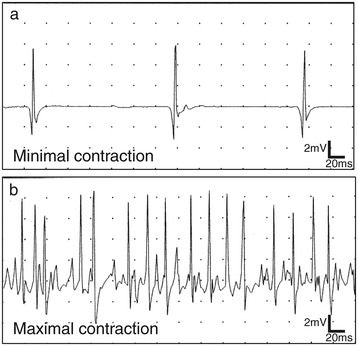
**Needle electromyograms of lower limbs.** Needle electromyograms show chronic neurological changes. High amplitude motor unit potential **(a)** and reduced interference pattern **(b)** in right tibialis anterior muscle are observed.

A motor conduction study of the tibial nerve showed low amplitude compound muscle action potential with no decrease in conduction velocity. F wave studies showed repeater F waves in median and tibial nerves, with low persistence. However, sensory nerve conduction studies of median, ulnar and sural nerves were normal. Needle electromyogram showed chronic neurogenic changes especially in the lower limbs (Table 1, Figure 2). These results strongly suggested degeneration of lower motor neurons.

**Figure 3:**
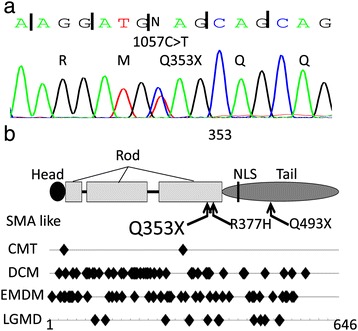
**Gene analysis of the** ***lamin*** **A/C gene (** ***LMNA*** **).** Sequencing of *LMNA* in our patient shows a heterozygous nonsense mutation (Q353X) in exon 6 **(a)**. Structure of lamin A and the positions of mutations in various laminopathies are shown **(b)**. For spinal muscular atrophy (SMA) phenotype, two of three mutations are located in the rod domain ([6] and this report). Diamonds indicate the mutations positions of laminopathies. The rod domain of lamin A is a hot spot for neuromuscular and cardiac diseases such as Charcot-Marie-Tooth disease type 2B1 (CMT2B1), dilated cardiomyopathy (DCM), Emery-Dreifuss muscular dystrophy (EDMD) and limb-girdle muscular dystrophy (LGMD).

With a presumptive diagnosis of SMA type 3, we conducted gene analysis. Exon 7 and 8 deletions of *SMN1* were not found. Early-onset muscular atrophy combined with cardiomyopathy made us to consider the possibility of inherited Charcot-Marie-Tooth disease (CMT) as a differential diagnosis. Some familial cases of SMA phenotypes with cardiac involvement were reported in some parts of Japan [5], and, within the responsible genes of CMT, *LMNA* mutations were reported to cause SMA phenotype with cardiac involvement in Germany [6]. For those reasons we added sequence analysis of *LMNA* gene, and a novel nonsense mutation p.Q353X (c.1057C > T) in exon 6 of *LMNA* was detected (Figure 3a). Exon 6 encodes the rod domain of lamin A and lamin C, which results in truncated lamin A protein lacking tail domain including nuclear localization signal (NLS) site (Figure 3b). The genetic analysis was limited to the proband, as we could not get a permission from other family members.

The molecular mechanisms of autosomal dominant SMA are largely unknown. However, Rudnik-Schöneborn et al. [6] reported adult-onset autosomal dominant proximal SMA phenotype with *LMNA* mutations in two families; one family with a novel nonsense mutation Q493X (c.1477C > T) and the other with missense mutation R377H (c.1130G > T) previously described in Emery-Dreifuss muscular dystrophy and limb-girdle muscular dystrophy 1B [7]. Their report indicates that *LMNA* mutations can mimic SMA and some patients have cardiac diseases such as atrioventricular block and DCM. The patients in the two families had onset of gait disturbance from early adulthood to maturity, with cardiac diseases diagnosed at the same time or several decades later. In one family, two of five cousins died from heart attack in their 40’s-50’s.

Our case is the second report of SMA phenotype with *LMNA* mutation. The patient showed muscle weakness of lower limbs since early childhood, and had cardiac diseases including atrial fibrillation, atrioventricular block and cardiac dysfunction in maturity. Amyotrophy was severe in proximal lower limbs and the trunk. This distribution of amyotrophy and findings of nerve conduction study are consistent with proximal SMA phenotype. Their clinical presentations were similar to previous cases family especially in cardiopathy, however, the gait disturbance were shown in different age. He had a novel nonsense mutation p.Q353X (c.1057C > T) in exon 6 of *LMNA* (Figure 3). In general, nonsense mutation in coding exon results in a significant molecular functional defect even with heterogeneity. The patient’s uncle (I-4, Figure 1) showed no muscular weakness, but died suddenly in his 20’s. According to a meta-analysis of carriers of *LMNA* mutations, sudden death was reported in 46% in both patients with cardiac diseases and those with neuromuscular diseases [8]. Electrocardiographic findings indicated cardiac dysrhythmias in 92% of the patients after the age of 30 years, while heart failure was reported in 64% after the age of 50 years. Furthermore, 28% of carriers of *LMNA* mutations were implanted a pacemaker [8].

*LMNA* spans 12 exons and is located on chromosome 1q21-22. It encodes lamin A and lamin C via alternative splicing. These lamins belong to the family of type V intermediate filaments. The protein consists of an N-terminal head domain, a central α-helical coiled coil rod domain, and a C-terminal tail domain (Figure 3b) [9]. Lamins are expressed exclusively in the nucleus of differentiated cells, and are the major constituents of the nuclear lamina and distributed in the nucleoplasm forming a part of the skeleton-type structure. Lamins also show high affinity to DNA, chromatin and histone for regulating gene expression.

*LMNA* mutations have been associated with several genetic disorders with different phenotypes and hereditary modes. These disorders are called laminopathies, and they include autosomal dominant Emery-Dreifuss muscular dystrophy (EMDM), autosomal repressive EMDM, Limb-girdle muscular dystrophy 1B (LGMD1B), Dilated cardiomyopathy with conduction defects (CMD1A), and CMT type 2B1 (CMT2B1). Laminopathies involving the muscular system and the peripheral nervous system both of which have mutations mainly at the N-terminal side of the NLS (Figure 3b). These diseases are suspected to result from abnormalities of the nuclear laminar structure. On the other hand, abnormality in genes encoding lamin-associated proteins such as *emerin* also caused EMDM [10], and the pathogenic mechanisms of laminopathies are not clarified. Several phenotypes of laminopathies were reported within the same family [10], and the interactions with other laminar factors might make part of the differences.

Distal hereditary motor neuropathies (dHMN), also referred to as distal SMA, manifest motor-dominant axonal neuropathy. This group shares several gene mutations with CMT2 [11,12]. Nevertheless there is no pathological evidence to differentiate whether our case is proximal SMA or dHMN, although electrophysiological findings and the distribution of amyotrophy suggest proximal SMA phenotype.

Two missense mutations of *LMNA*; E33D and R298C, have been reported in CMT2B1 [13,14]. They are associated with abolished deep tendon reflex and amyotrophy mainly affecting lower limbs. A combined phenotype with cardiac complications such as arrhythmia and cardiomyopathy is possible [13]. Interestingly, some cases of CMT2B1 due to *LMNA* mutations show amyotrophy in proximal limbs [13]. In these cases, degeneration of neurons in the anterior horn of spinal cord is suspected. The mechanisms underlying neuropathy and neuronopathy of lower motor neurons may be different.

## Conclusions

We report a novel *LMNA* mutation in a patient with juvenile-onset SMA-like gait disturbance, combined with cardiac diseases including atrioventricular block and cardiac hypofunction with family history of sudden death. The previous report indicating the nonsense and missense *LMNA* mutation can induce SMA phenotype with cardiomyopathy [6] ensures our similar phenotype case with novel nonsense *LMNA* mutation is worth reporting even though genotype-phenotype cosegregation could not be demonstrated due to lack of family samples. In cases of SMA clinical presentation/phenotype without *SMN1* deletion, careful cardiac work-up should be conducted and *LMNA* gene analysis should be considered especially when the family history is compatible with a neuromuscular disease and/or an unexplained cardiopathy and/or when sudden death are present.

## Consent

Written informed consent was obtained from the patient for publication of this case report. Additionally, informed consent was obtained for genetic analysis. A copy of the written consent is available for review by the Editor of this journal.

## Abbreviations

SMA: Spinal muscular atrophy
*SMN1*: Survival motor neuron gene
*LMNA*: *Lamin A/C* gene
DCM: Dilated cardiomyopathy
NLS: Nuclear localization signal
CMT: Charcot-Marie-Tooth disease
EMDM: Emery-Dreifuss muscular dystrophy
LGMD1B: Limb-girdle muscular dystrophy 1B
CMD1A: Dilated cardiomyopathy with conduction defects
CMT2B1: Charcot-Marie-Tooth disease type 2B1
dHMN: Distal hereditary motor neuropathies
